# Immigration policy changes and impact on the mental health of immigrants

**DOI:** 10.1192/j.eurpsy.2026.11636

**Published:** 2026-07-31

**Authors:** E. C. Ezema, A. Meftah, B. Aribisala, T. Htet, E. U. Ezenagu, S. Singh, T. Sultana, J. Beauchamp, T. Olupona

**Affiliations:** Psychiatry, One Brooklyn Health-Interfaith , Brooklyn, United States

## Abstract

**Introduction:**

Immigration policies have a profound effect on the mental health of immigrants by fostering atmospheres of fear, uncertainty, and stress, which can intensify issues such as anxiety, depression, and Post Traumatic Stress Disorder (PTSD). Modifications in policy may limit access to education, employment, and healthcare, while threats of deportation and family separation contribute to cumulative social disadvantages that deteriorate mental well-being, underscoring a vital connection between immigration legislation and the psychological condition of immigrants.

**Objectives:**

To evaluate how immigration policy changes result in anxiety, depression, and PTSD among the immigrants

**Methods:**

We conducted a systematic review using search terms “immigration policy changes and migrants ‘mental health,” “immigration policy and psychological distress of migrants,” “immigration policy and migrants’ challenges”. The search was conducted on Google Scholar, PubMed, and the Bielefeld Academic Search Engine. It yielded 15 articles.

**Results:**

From the survey of 58,087 participants in a study in the UK, greater psychological distress was observed among the minoritised ethnic immigrants. Of the other articles, 50 % reported depressive symptoms. Anxiety was reported in ten articles, while many met criteria for PTSD.

Mental health issues such as depression, anxiety, and PTSD are documented in adult undocumented immigrants, as well as in undocumented children. These children endure considerable trauma, which can lead to the emergence of PTSD symptoms within this vulnerable population. Furthermore, undocumented children encounter distinct obstacles, including educational barriers and anxiety regarding the arrest, incarceration, and imprisonment of family members due to their immigration status, resulting in heightened trauma and distress.

**Conclusions:**

The health risks faced by immigrants are exacerbated by immigration policies and laws that foster a heightened fear of detection, create language barriers, and contribute to the deterioration of mental health. A global strategy is required to address the challenges posed by immigration and unauthorized cross-border movements. It is essential for healthcare professionals, politicians, stakeholders, think tanks, advocacy organizations, and others to unite and formulate policy solutions grounded in the principles of social justice and human rights

**Disclosure of Interest:**

None Declared

